# Effects of compost as a soil amendment on bacterial community diversity in saline–alkali soil

**DOI:** 10.3389/fmicb.2023.1253415

**Published:** 2023-09-27

**Authors:** Daolong Xu, Xiaowen Yu, Jin Chen, Xiufen Li, Jian Chen, JiangHua Li

**Affiliations:** ^1^National Engineering Laboratory for Cereal Fermentation Technology, Jiangnan University, Wuxi, Jiangsu, China; ^2^Key Laboratory of Forage and Endemic Crop Biotechnology, Minister of Education, School of Life Sciences, Inner Mongolia University, Hohhot, China; ^3^College of Life Sciences, Anhui Agricultural University, Hefei, China; ^4^School of Environment and Civil Engineering, Jiangnan University, Wuxi, China

**Keywords:** composting, saline-alkali stress, high-throughput sequencing, soil bacterial community, physical and chemical properties, soil amendment

## Abstract

**Introduction:**

Soil salinization poses a worldwide challenge that hampers agricultural productivity.

**Methods:**

Employing high-throughput sequencing technology, we conducted an investigation to examine the impact of compost on the diversity of bacterial communities in saline soils. Our study focused on exploring the diversity of bacterial communities in the inter-root soil of plants following composting and the subsequent addition of compost to saline soils.

**Results:**

Compared to the initial composting stage, Alpha diversity results showed a greater diversity of bacteria during the rot stage. The germination index reaches 90% and the compost reaches maturity. The main bacterial genera in compost maturation stage are *Flavobacterium*, *Saccharomonospora*, *Luteimonas* and *Streptomyces*. Proteobacteria, Firmicutes, and Actinobacteria were the dominant phyla in the soil after the addition of compost. The application of compost has increased the abundance of Actinobacteria and Chloroflexi by 7.6 and 6.6%, respectively, but decreased the abundance of Firmicutes from 25.12 to 18.77%. Redundancy analysis revealed that soil factors pH, solid urease, organic matter, and total nitrogen were closely related to bacterial communities.

**Discussion:**

The addition of compost effectively reduced soil pH and increased soil enzyme activity and organic matter content. An analysis of this study provides theoretical support for compost’s use as a saline soil amendment.

## Introduction

Saline land refers to soil with high levels of salinity and alkalinity, which are harmful to plant growth and agricultural production (Yin et al., 2022). Soil saline–alkalinization is a global problem, especially in coastal and semiarid areas. Approximately 1 billion hectares of salinized soil cover the world’s arable land, which is increasing at a rate of 2% a year (He et al., 2020; Cui et al., 2021). The main characteristic of saline land is the high level of salinity and alkalinity in the soil, which severely affects plant growth. When salts accumulate to a certain level in the soil, they can poison the roots of plants and affect their absorption of nutrients and water, eventually leading to slow growth or even death (Ali et al., 2017). Saline soils may also decrease soil quality, biodiversity, water content, and oxygen content, thereby affecting agricultural production and the ecological environment (Setia et al., 2013). Three key attributes of saline soils that impede plant growth and diminish agricultural productivity encompass elevated salinity levels, deterioration of soil structure, and nutrient depletion (Liu et al., 2021). Hence, enhancing the condition of saline land is deemed crucial not only for safeguarding the ecological environment and agricultural productivity, but also as an imperative approach to augment agricultural efficacy and enhance the quality of human life. Solving the problem of soil salinization is also significant for protecting the ecological environment, promoting agricultural development, and improving human quality of life (Chen et al., 2021; Yang et al., 2022).

Composting is an effective biological method to improve soil texture and crop yields (Shen et al., 2019). Waste is converted into humus material using microorganisms, which can be transformed into fertilizer and soil conditioner through organic waste treatment and compost production (Qayyum et al., 2017). Using green organic amendments (compost products, such as plant and animal fertilizers, sludge, and food and agricultural wastes) to repair soils can improve their fertility. These soil amendments significantly improve the biological properties and soil organic matter (SOM) of saline–alkali soil (Chavez-Garcia and Siebe, 2019). Research has demonstrated that the application of biochar compost to saline soils can enhance various physicochemical attributes, including soil organic matter (SOM) and nutrient content, consequently fostering plant growth and augmenting crop yields (Zheng et al., 2018; Liang et al., 2021). Aiad et al. (2021) found that restoration of saline soils using soil amendments (i.e., compost and zeolite) significantly reduced soil salinity and increased wheat and corn yields by 16.0 and 35.0%, respectively. A recent study has shown that by increasing the addition rate of cow manure compost, corn yields can be increased by 6.0–28.4% (Li et al., 2022). Nevertheless, there remains a need for further investigation to comprehensively elucidate the effects of compost amendment on the microbial community structure in saline-alkali soil, as well as to establish a clearer understanding of the intricate relationship between microbial communities and environmental factors.

As a key component of soil ecosystems, microorganisms are widely considered important indicators of soil quality (Joergensen and Wichern, 2018). Soil microorganisms play a crucial role in the processes of material and energy cycling, soil structure maintenance, and soil microecological balance within the soil. During composting, microorganisms facilitate the decomposition, transformation, and synthesis of organic matter (Wang S. P. et al., 2022). Composting microorganisms mainly include bacteria, fungi, protozoa, and viruses (Duan et al., 2022). These microorganisms are mainly obtained from the starting composting material and recruited from the composting environment (Wei et al., 2018). With the decomposition of organic materials at each stage, various biochemical indicators in the composting system keep changing, and different microorganisms alternately dominate, independently or in cooperation with other microorganisms, to decompose and transform organic materials and promote compost decomposition (Singh and Nain, 2014). Zhao et al. (2022) found that Oceanobacillus, Bacillus, Pseudogracilibacillus, and Nocardiopsis were mainly present in sheep manure compost; At different stages, bacterial communities differed in abundance. Additionally, Neher et al. (2013) discovered that composting microorganisms exhibit associations with various materials, methods, and stages employed in the composting process. Furthermore, it has been documented that microbial communities exhibit responses to various external environmental stimuli, including temperature fluctuations, compost composition, and carbon-to-nitrogen ratios (C/N; Chen et al., 2019). However, the predominant focus of previous research has been on the analysis of changes in microbial community dynamics during the composting process. There has been a limited number of investigations that have explored the effects of compost application with different materials on the microbial composition in saline soils, along with its correlation with environmental factors. The primary aim of this study was to investigate the influence of compost application as a soil amendment on the composition and functionality of microbial communities in saline soils.

The utilization of high-throughput sequencing (HTS) technology has proven to be an efficient method for detecting alterations within microbial communities. This technology is extensively employed in the examination of intricate microbial communities inhabiting soil across various habitats. Therefore, in this study, we performed composting and potting experiments and hypothesized that compost addition could improve saline soils by changing the soil microenvironment. The aim of this study was to examine the influence of different levels of saline-alkali soil on bacterial communities throughout the composting process, employing high-throughput sequencing. Moreover, the primary objective of this study was to evaluate the efficacy of compost application in augmenting plant growth and ameliorating soil quality. Additionally, the study aimed to investigate the impact of compost application on the composition of bacterial communities in saline soils, along with their correlation with environmental factors.

## Materials and methods

### Materials

Saline soils collected from Zhenlai County, Baicheng City, Jilin Province (122°47′–124°04′E, 45°28′–46°18′N), were prepared by Jiangsu Guoxin Xielian Energy Co. Using a 2-mm sieve, the saline soils were mixed evenly after air-drying. The experimental crop was radish (Fengguang Generation), and the experiment began in the laboratory in January 2022. Sheep manure and corn straw were purchased separately from the market. Saline soils had the following initial properties: total nitrogen (TN): 1.39 g kg^−1^, total carbon: 7.02 g kg^−1^, SOM: 2.84 g kg^−1^, pH: 10.74, and electrical conductivity (EC): 1.36 dS m^−1^ (Table 1).

**Table 1:** Basic properties of raw materials.

Composting materials	*w* (C)/%	*w* (N)/%	C/N	Moisture (%)	pH
Sheep dung	25.60 ± 0.86	2.07 ± 0.10	12.36 ± 0.36	10.43 ± 070	8.30 ± 0.48
Rice straw	41.37 ± 0.90	1.60 ± 0.05	25.91 ± 0.31	10.40 ± 0.59	6.8 ± 0.22
Saline-alkali soil	7.02 ± 0.22	1.39 ± 0.15	5.11 ± 0.61	6.49 ± 0.37	10.74 ± 0.04

Values are presented as mean ± standard deviation (*n* = 3).

### Aerobic composting procedure

The reactor for composting was a 75-cm × 55-cm × 60-cm polypropylene plastic box with a total reactor volume of 247.5 L. During composting, porous sieve plates were placed 10 cm from the bottom of the plastic box. Aeration was maintained throughout the composting process at 0.2 L/(L min). Three different proportions of saline soil were used in the composting process (MA: 0.8 kg, MB: 4 kg, and MC: 8 kg) mixed mechanically with straw and sheep manure. The total mass and volume of saline soil, sheep manure and straw in each reactor were 32 kg and 240 L. We mixed sheep manure with corn straw in a ratio of 1:2 by dry weight, with a C/N of approximately 25. Composting was adjusted at 60% soil moisture content (SMC; Wu et al., 2017). The material was turned and mixed well at regular intervals (days 0, 2, and 5 and then weekly). Table 1 shows the properties of the raw materials. The composting process was divided into four stages based on temperature variations: (S) warming period (day 0), (G) high-temperature period (days 1–5), (J) cooling period (days 6–10), and (F) decaying period (days 11–30). A portion of the soil samples was stored at 4°C for physicochemical analysis, while the other portion was stored at −80°C to facilitate microbial analysis.

### Pot experiments

Pot experiments were conducted using control (CK) and three treatments (MA, MB, and MC compost addition), with four replicates per treatment, obtaining a total of 16 pots. Each pot, measuring 19 cm in diameter and 17 cm in depth, was filled with 0.8 kg of substrate and subsequently planted with a total of 20 radishes. The substrate consisted of saline soil and compost (1,3). For CK, no compost was added to the saline soil; for AP, MA compost product was added; for BP, MB compost product was added; and for CP, MC compost product was added. The pots were irrigated by hand until they reached 60% of their water holding capacity. In addition, roots are collected from potted plants, soil is stripped, and ~1 mm of soil is left around the roots. Subsequently, ~1 mm of soil was washed away in the PBS buffer, and 4 composite samples were prepared from each group of treated soil. The collected soil samples were subsequently partitioned into two segments: one segment was preserved at a temperature of 4°C for physicochemical analysis, while the other segment was preserved at a temperature of −80°C for microbial analysis.

### Physicochemical parameter analysis

The pH of all fresh soil samples was determined by air-drying, grinding, and passing them through a 2-mm sieve; temperature (T); EC; and SMC, TN, sodium (Na), solid catalase (SCAT), and SOM contents. A fully automatic Kjeldahl nitrogen tester (Foss, NKY6120, Germany) was used to determine the TN content of the soil. The pH and electrical conductivity (EC) were determined by employing a pH electrode (HM-25G, TOA DKK, Japan) and an EC indicator (SG3, Mettler Toledo, United States), respectively. The measurement of SOM content was conducted through the utilization of the muffle furnace scorch method, while the determination of soil potassium (K) and sodium (Na) contents was carried out using a flame photometer (FP640; Li et al., 2021). Soil enzyme activity was determined using a soil enzyme kit. In the conducted seed germination experiments, a water extract was utilized. Specifically, a total of 10 radish seeds were evenly distributed onto a filter paper and subsequently subjected to incubation in darkness at a temperature of 20°C for a duration of 48 h. Three replicates of each compost sample were individually analyzed, with each treatment being assessed through the quantification of germinated seeds and the measurement of root length. The germination index (GI) was calculated as follows:

$\text{GI}\;\left(\%\right)=\frac{\text{Seeds germinated in the extract}\;\left(\%\right)\cdot \times \text{Root length of treatment}}{\text{Seeds germinated in the extract}\;\left(\%\right)\cdot \times \cdot \text{Root length of control}}\times 100$

### Microbial community analysis

#### DNA extraction and detection

The Power Soil DNA extraction kit (MoBio Laboratories, Inc., Carlsbad, CA, United States) was employed to extract the complete genetic DNA from compost and potted soil samples. Compost and potting soil samples in triplicate and then combined. The quality and quantity of extracted DNA was assessed using the NanoDrop 1000 spectrophotometer (Thermal Sciences, Wilmington, DE, United States). The polymerase chain reaction (PCR) was conducted using TransGen AP221-02: TransStart Fastpfu DNA polymerase (20 μL) along with 0.8 μL (5 μM) of both forward and reverse primers, 10 ng of template, 4 μL of buffer (5×), 2 μL of deoxynucleoside triphosphates (2.5 mM), 0.4 μL of polymerase, and 0.2 μL of bovine serum albumin. After detection on 2% (w/v) agarose gel, the final amplicon was quantified using the AxyPrep DNA gel extraction kit (Axygen Biosciences, Union City, United States). Using the Yili MiSeq platform, the same amount of purified amplicon was collected at Alway Gene (Beijing, China) for follow-up sequencing. The region of the bacterial 16S rRNA gene, specifically 338F_806R, was amplified using primers 338F (5-ACTCCTACGGGAGGCAGCAG-3) and 806R (5-GGACTACHVGGGTWTCTAAT-3; Xu et al., 2020). The processes of PCR amplification, library preparation and detection, and computer sequencing analysis were conducted by Shanghai Meiji Biomedical Technology Co., Ltd. The Illumina sequences were deposited in the NCBI Sequence Read Archive (accession numbers SRR24001333–SRR24001347 for the rhizosphere soil bacteria of potted 16S rRNA gene data and SRR24031951– SRR24031993 for the compost rhizosphere soil 16S rRNA gene data).

### Bioinformatics analysis

After performing sample splitting of PE reads acquired through Illumina sequencing, the data was optimized by subjecting it to quality control (QC) splicing. This involved initially conducting QC procedures and subsequently filtering the double-ended reads based on sequencing quality. Additionally, the splicing process was carried out by considering the overlapping relationship between the double-ended reads. The optimized data underwent processing utilizing sequence noise reduction techniques, such as DADA2/Deblur, in order to acquire representative sequences and abundance information for amplicon sequence variants (ASVs). Using ASV representative sequences and abundance information, various statistical and visual analyses can be conducted, encompassing species taxonomy, community diversity, species dissimilarity, correlation, phylogenetic, and functional prediction analyses.

### Statistical analysis

For the multivariate statistical analysis (correlation analysis), SPSS version 19.0 was used. A significance threshold of 0.05 was used to determine statistical significance. Data were collected in four replicates and subjected to analysis of variance (ANOVA). Principal component analysis (PCA) was employed to evaluate variations in bacterial and fungal communities, as well as their transformations throughout the composting process. The application of redundancy analysis (RDA) unveiled the correlation between environmental factors and the manifold alterations in bacterial community composition (Chen et al., 2020). The R heat map package (version 3.3.1) was employed for the production of heat maps. The sample distance matrix was subjected to clustering and analysis, with the R language employed for constructing the dendrogram. Majorbio I-Sanger was used for all bioinformatics analyses.

## Results and discussion

### Evolution of physicochemical properties during composting

**Figure 1:**
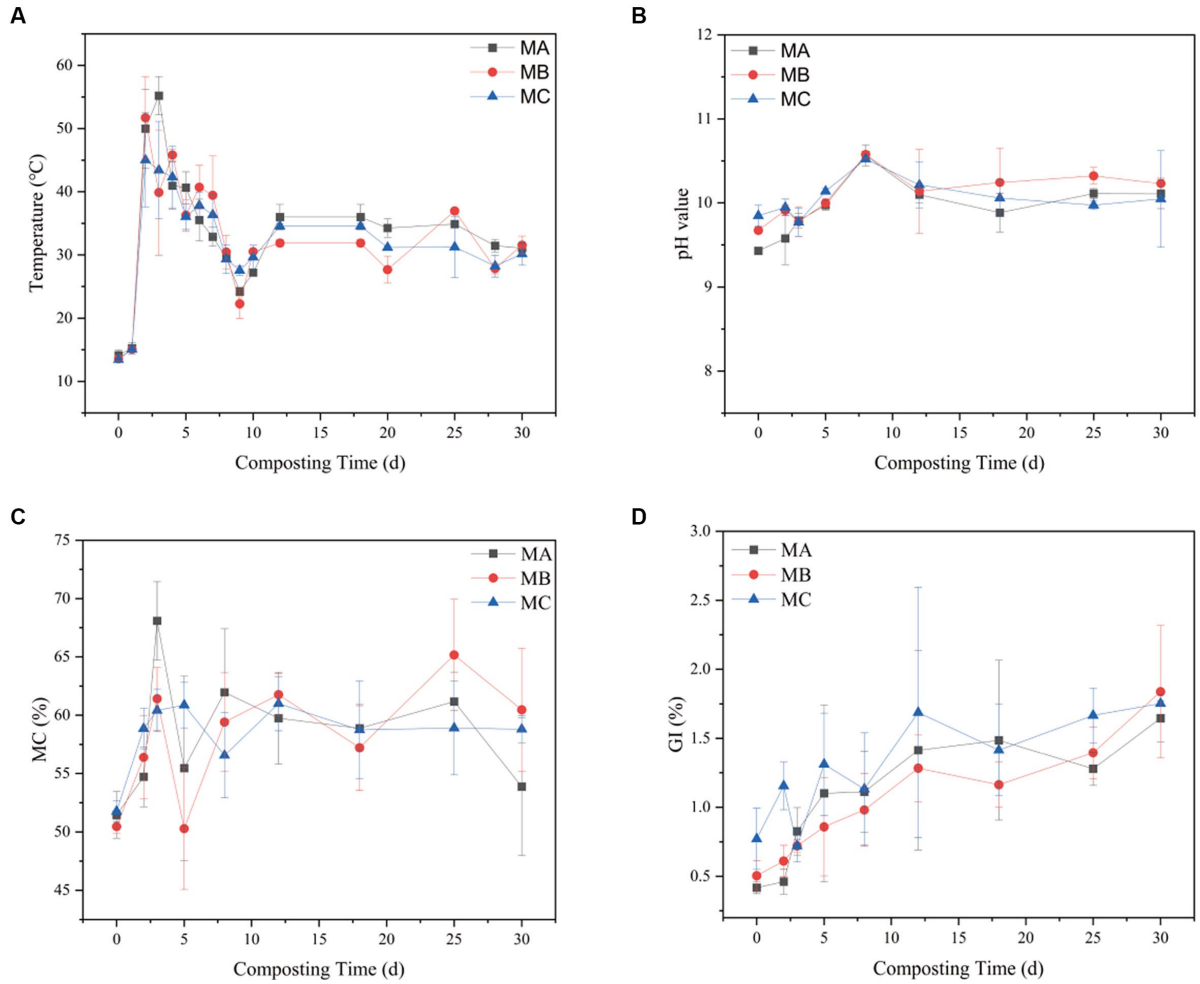
Changes in **(A)** temperature, **(B)** pH, **(C)** SMC, and **(D)** GI during composting in the three treatments.

The temperature, pH, soil moisture content (SMC), and germination rates of the compost were assessed at various time points. It was observed that temperature played a crucial role in influencing the decomposition of organic matter and microbial activity throughout the composting process. Figure 1A illustrates that the temperature fluctuations were consistent across the three treatment groups, which underwent four distinct stages of composting: warming, high-temperature, cooling, and decaying periods (Ren et al., 2018). There was a significant difference in the rate of temperature change among the three treatments. The temperatures of MA and MB rose to a peak of around 55C within a span of 2 to 3 days, surpassing the rate of warming observed in MC. This observation implies that the rate at which organic constituents degrade increases as compost maturity increases, possibly due to the enhancement of microbial community diversity during the composting process (Kato and Miura, 2008). The higher the content of saline-alkali soil, the slower the temperature rise of compost. As the composting carbon source was consumed, the composting temperature decreased until a stabilization period was reached around day 12. The trend in pH changes was similar for all composting processes (Figure 1B). However, the higher the content of saline-alkali soil, the greater the pH change during composting. The pH started to increase significantly on day 2, possibly due to ammonia emission and organic acid depletion; this discovery aligns with the research conducted by Mei et al. (2021). On day 8, a decline in pH was observed as a result of microbial activity leading to the generation of small-molecule organic acids that rapidly decompose within the composted material. After day 18, the pH eventually leveled off as the compost reached the decomposition stage, forming large amounts of humus, followed by slight fluctuations.

SMC was also monitored at different times. MA had the highest average SMC of 69%, followed by MB with 62%, and the overall SMC reached >50% (Figure 1C). In the high-temperature stage, the microbial metabolism led to a large amount of water evaporation. MA, MB, and MC decreased by 22.15, 12.32, and 10.10%, respectively. These changes indicated that some microorganisms in different proportions of saline–alkali soil play an important role in fermentation, similar to a previous study (Guo et al., 2015). The maturity and phytotoxicity of the compost were assessed using the germination index (GI). Upon reaching a GI of 90%, the compost demonstrated the loss of phytotoxicity and achieved maturity (Figure 1D). In the early stages of composting, the glycemic index (GI) of each group exhibited a relatively low value, which could be potentially ascribed to the existence of NH3 and fatty acids generated through the swift breakdown of organic matter (Jiang et al., 2018).

### Bacterial community profiles in composting systems

**Figure 2:**
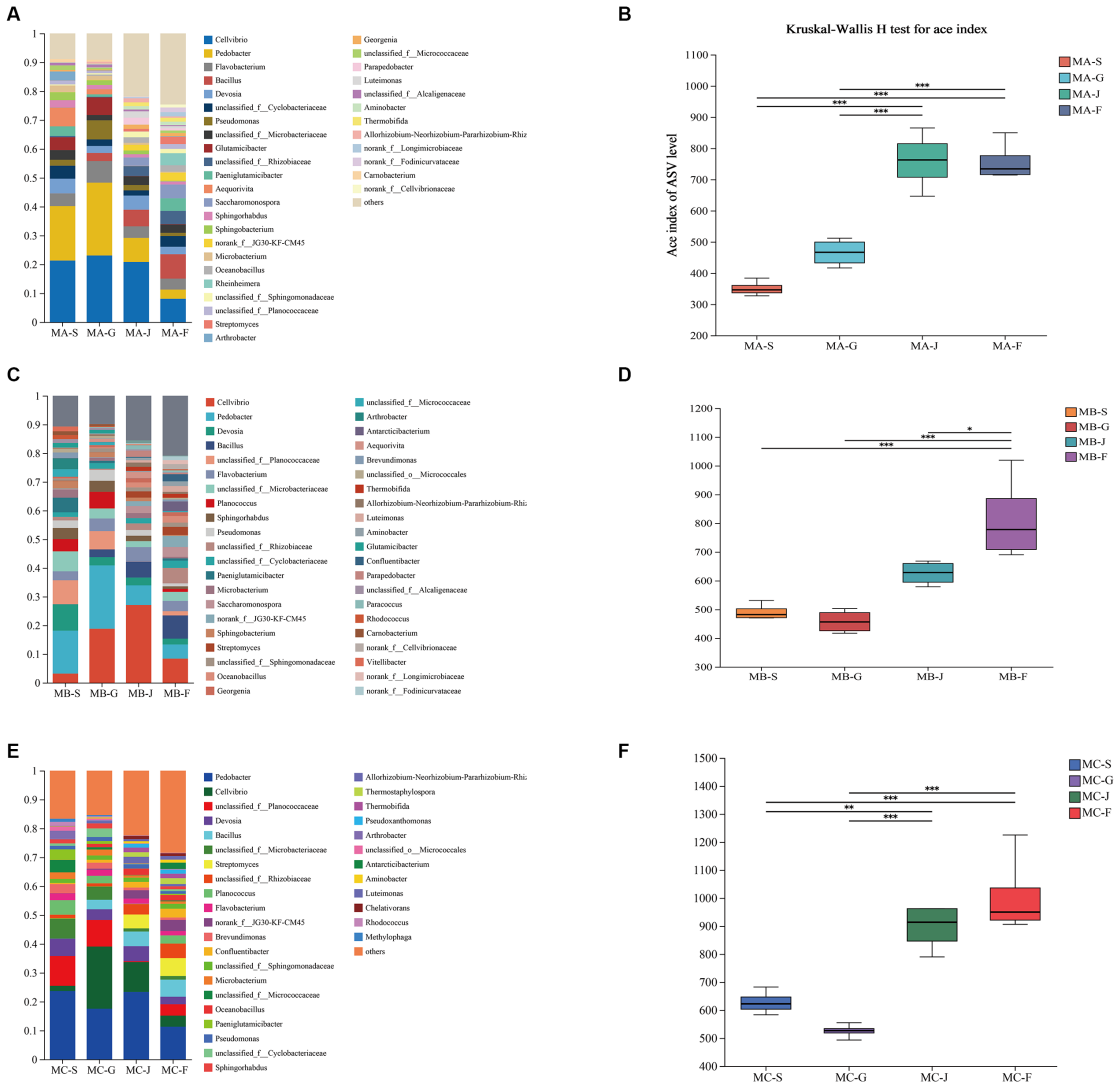
Evolution of bacterial community characteristics in different composting stages of the three treatments. **(A,C,E)** Relative abundance of bacteria at the genus level. Horizontal/vertical coordinates are sample names, vertical/vertical coordinates are the proportion of species in that sample, different colored bars represent different species, and the length of the bar represents the size of the proportion of that species. **(B,D,F)** Bacterial α-diversity. Significant differences between different samples mark the two groups with significant differences (0.01 < *p ≤ 0.05, 0.001 < **p ≤ 0.01, ***p ≤ 0.001).

The modulation of composting process and product quality is significantly influenced by alterations in bacterial communities, as demonstrated by Wang X. H. et al. (2022). Figure 2A illustrates the noteworthy temporal dynamics observed in the genus-level bacterial community composition across different treatments throughout the composting progression. In the warming period of composting (S), *Cellvibrio*, *Pedobacter*, *Devosia*, *Planococcus*, *Planococcus*, and *Microbacterium* were the dominant genera. The findings of this study align with the outcomes of a prior investigation pertaining to the fluctuations in bacterial community dynamics throughout the composting process of distilled grain waste (Wang et al., 2022a). As composting proceeded into the high-temperature period, *Pedobacter*, *Pseudomonas*, and *Cellvibrio*, belonging to Bacteroidetes and Proteobacteria phyla, increased greatly and became the dominant genera. During the progression of composting into the thermophilic phase (MA-G, MB-G, and MC-G), there was a significant increase in the abundance of the Pedobacter genus, which emerged as the predominant genera, constituting 25.2, 22, and 17.56%, respectively, of the overall bacterial community (Figures 2A,C,E). In MA-G, MB-G, and MC-G, compared with the warming period of composting, Actinobacteriota abundance decreased significantly. During the cooling phase, there was a notable similarity in the bacterial community structure observed among MA, MB, and MC; however, the abundance was different. In comparison to the thermophilic phase, it was observed that the relative abundance of Pedobacter decreased from 25.2 to 8.32% in MA and from 22.06 to 6.86% in MB. Furthermore, there was a notable increase in the prevalence of Bacillus in the composts, with percentages rising from 2.79 to 8.44% in MA and from 2.70 to 8.06% in MB (Figures 2A,C). The findings of this study exhibited a resemblance to those of a prior investigation (Wang S. et al., 2020), indicating that microbial community succession is related to compost temperature. Proteobacteria, Bacteroidetes, and Actinobacteria are the predominant phyla of the three groups. Prior research has similarly documented that these bacteria exhibit the highest prevalence in saline-alkali soil (Wang S. et al., 2020; Nan et al., 2022). Intriguingly, during the decay phase, there was a notable decrease in the abundance of Bacteroidetes and Actinobacteria, while the presence of Proteobacteria exhibited a significant increase. These findings suggest that the development of fully mature compost has a discernible impact on the progression of bacterial communities. Proteobacteria assume a significant function in the process of nutrient recycling (nitrogen and carbon) and decomposition of organic matter (Wan et al., 2021). The increase in Proteobacteria increased the nutrient level in saline–alkali soil and decreased soil alkalinity. Furthermore, Proteobacteria, a group of thermophilic heterotrophic bacteria, have the ability to augment the process of cellulose degradation in composting (Zhang et al., 2020). Among them, *Flavobacterium*, *Saccharomonospora*, *Luteimonas*, and *Streptomyces* mainly appeared in the decaying period. These bacteria exhibited a positive influence on plant growth and demonstrated the ability to suppress the proliferation of soil-borne diseases, aligning with prior research findings (Andric et al., 2020; Ding et al., 2021), indicating that three different compost treatments have finally reached maturity. Hence, the agricultural suitability of the products derived from all three composting processes is evident.

By calculating the alpha-diversity index, the ACE indices of the three compost treatments at different stages (warming, high-temperature, cooling, and decaying periods) were compared. ANOVA revealed significant changes in bacterial alpha-diversity at different stages of composting (Figures 2B,D,F). The bacterial community diversity trend was similar among the three treatments at the mature stage of compost, and the bacterial community diversity at the rot stage was higher than that at the initial stage of compost. The findings of this study indicate that the process of composting resulted in a significant increase in the bacterial diversity of saline-alkali soil-straw-sheep manure compost. This increase in bacterial diversity is considered advantageous for the enhancement of saline-alkali soil quality (Shi et al., 2020; Wu et al., 2021). In the high-temperature period, the alpha-diversity index of MA, MB, and MC was lower than that of other periods, probably because the metabolism of thermophilic microorganisms was enhanced with the increase in temperature, resulting in the decreased diversity of other bacteria (Zhang et al., 2021). Hence, the genus species exhibited no notable disparity across various composting stages; however, the variability in genus abundance varied among distinct composting processes.

### Effects of compost products on the bacterial community structure in saline–alkali soil

**Figure 3:**
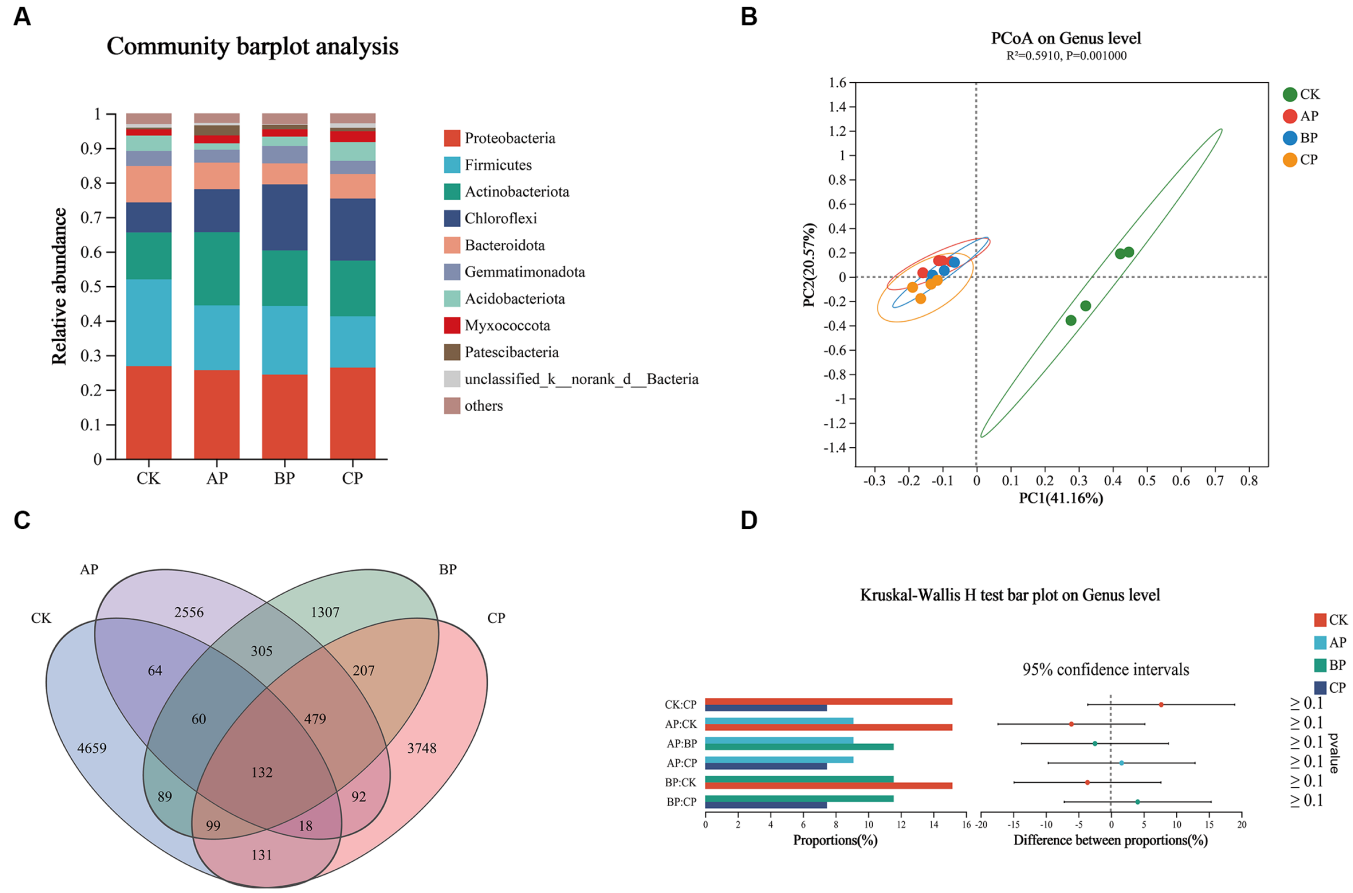
Effects of composting product on the bacterial community in saline–alkali soil plant with pot. **(A)** Relative abundance of bacteria at the phylum level. **(B)** PCA of bacterial community composition based on Bray–Curtis similarity. **(C)** Venn diagram showing the shared bacterial ASVs. **(D)** Analysis of species differences based on the Kruskal–Wallis H test. The horizontal coordinate of the bar graph on the left shows the percentage of *Bacillus* abundance in each group, and the ordinate shows the group type compared in pairs. The figure on the right shows the proportion of differences in species abundance.

The diversity of bacterial communities is of utmost importance in soil ecosystems, as it is crucial for understanding the structural characteristics of microbial communities (Zhou et al., 2020). The current investigation observed a significant influence of compost products on the bacterial community’s composition and diversity in the rhizosphere soil of plants (Figure 2). Examination at the phylum level indicated that Proteobacteria, Firmicutes, Actinobacteria, and Chloroflexi were the dominant phyla in the rhizosphere soil (Figure 3A), aligning with previous studies and suggesting a substantial increase in these bacterial taxa in saline-alkali soil (Wang et al., 2022b; Yuan et al., 2023). Moreover, in these saline-alkali soils, Proteobacteria exhibit a significant prevalence, constituting 12.81, 25.62, 24.39, and 26.38% of the bacterial community in the control, MA, MB, and MC groups, respectively. Prior research has demonstrated that Proteobacteria possess the capability to degrade diverse macromolecules and facilitate the cycling of carbon, nitrogen, sulfur, and other essential substances. Additionally, they play a crucial role in mitigating abiotic stress through nitrogen fixation and promoting plant growth (Bruto et al., 2014). Therefore, Proteobacteria can be applied in agricultural production as biological control agents in soil–plant ecosystems (Hu et al., 2022). In the current investigation, the application of compost resulted in a notable increase in the abundance of Actinobacteriota and Chloroflexi, while concurrently causing a significant decrease in the abundance of Firmicutes when compared to the control group. Actinobacteriota exhibit a pivotal function in the process of global soil decomposition and carbon cycling, which demonstrates an inverse relationship with salt stress. Consequently, an augmentation in carbon cycling is anticipated to alleviate the detrimental effects of salt stress (Upchurch et al., 2018). Chloroflexi also positively affected plant growth by promoting nitrogen absorption (Hua et al., 2021). Furthermore, it has been observed that the saline-alkali soil of Songnen Plain in northeast China harbors a substantial population of Firmicutes. Both Firmicutes and Proteobacteria have been recognized as bacterial taxa with the ability to withstand and mitigate saline-alkali stress (Wang Y. et al., 2020).

The effects of different proportions of compost products mixed with saline–alkali soil on microbial community diversity in plant rhizosphere soil were studied via PCA based on the bacterial level (Figure 3B). The findings indicated that the bacterial communities in the rhizosphere soil of the three treatments exhibited a similar distribution along the axis, suggesting that the application of varying proportions of compost products had minimal impact on the bacterial communities (Wu et al., 2017). The bacterial ASV numbers in CK, AP, BP, and CP were 4,659, 2,556, 1,307, and 3,748, respectively (Figure 3C). There was a significant disparity in the quantity of bacterial ASVs observed in saline-alkali soil with compost products compared to the control group. The examination of discrepancies between the groups revealed a notable distinction in the genus Bacillus between the control group and the other three groups at the genus level (Figure 3D). According to Khan et al. (2019), a prior investigation demonstrated the significant involvement of Bacillus in the metabolic processes of carbohydrates within the soil environment, as well as its ability to secrete plant hormones, thereby influencing the growth and development of plants. These findings suggest that the introduction of compost products alters the composition of the microbial community in soil, potentially leading to the presence of beneficial bacteria that can mitigate saline-alkali stress and enhance soil nutrient levels.

### Correlation among bacterial communities with physicochemical factors

**Figure 4:**
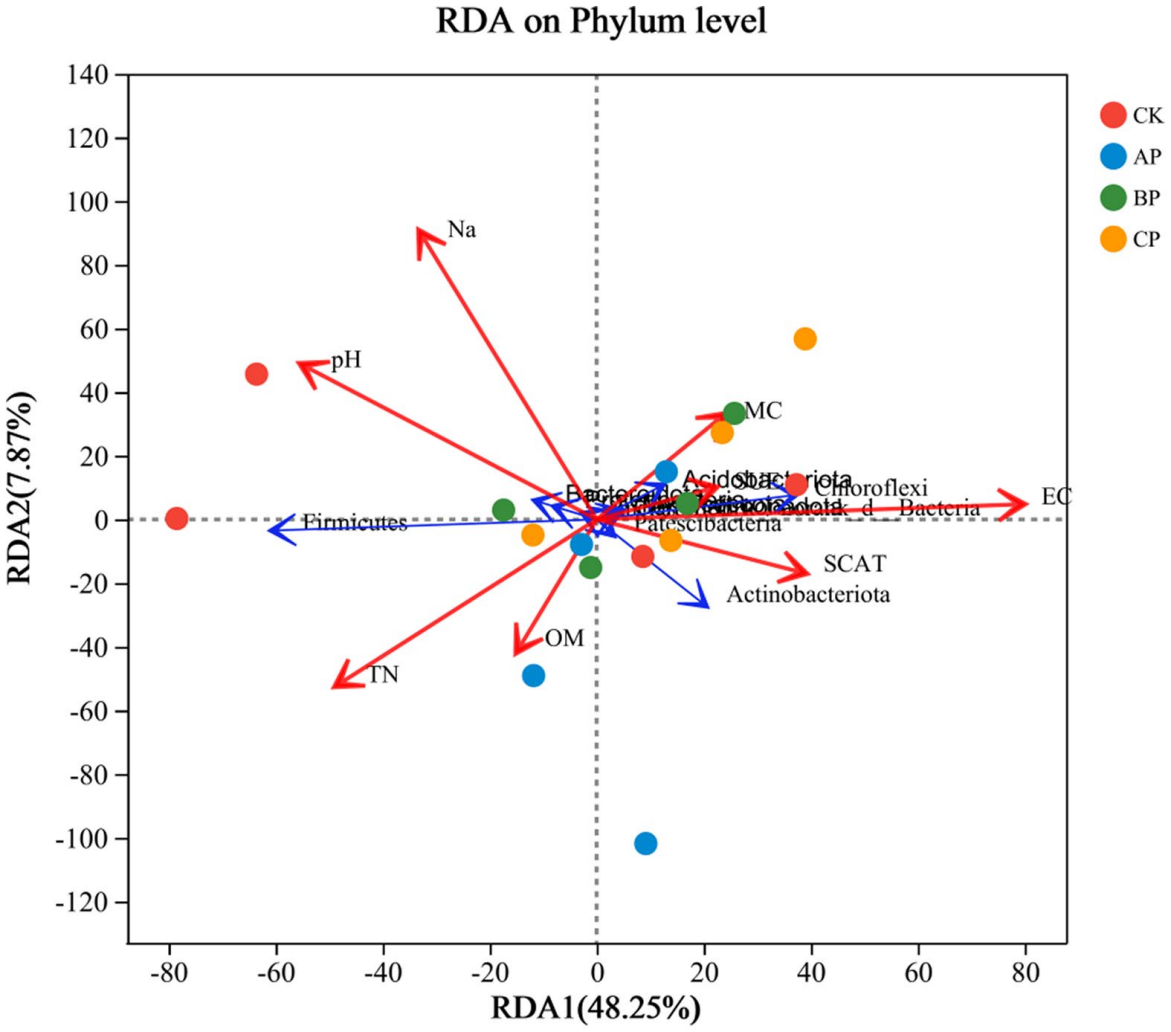
RDA of soil chemical properties and relative abundance of bacterial taxa. Red arrows represent the environmental factors (soil properties), and blue arrows represent the top 10 bacterial phyla in terms of relative abundance. TN, total nitrogen; OM, organic matter; EC, electrical conductivity; MC, moisture content; SCAT, solid catalase; SUE, solid urease.

Canonical correspondence analysis (CCA) was employed to investigate the correlation between the diversity of bacterial communities and the physicochemical characteristics of soil in the inter-rhizosphere soil of plants subsequent to the application of compost products in saline soils refer to Figure 4. The application of compost exhibited a significant and positive correlation with soil total nitrogen (TN), organic matter (OM), soil moisture content (SMC), electrical conductivity (EC), solid urease (SUE), and soil catalase (SCAT). Conversely, it displayed a negative correlation with soil sodium (NA) and pH. In general, soil pH, TN, SCAT, and EC were identified as the primary factors influencing the composition and diversity of the bacterial community. The salinity of saline soils leads to soil nutrient deficiency and affects plant growth. Compost application alleviated soil salinity and pH (Supplementary Figure S1A). The abundance of Proteobacteria, Firmicutes, Actinobacteriota, and Bacteroidota exhibited a significant correlation with soil physicochemical properties. Furthermore, Actinomycetes and Chloroflexi demonstrated a positive association with electrical conductivity (EC) and catalase, while displaying a negative relationship with pH. The study revealed that the association between the relative abundance of Chloroflexi and EC was not statistically significant. However, a positive correlation was observed between Chloroflexi abundance and pH and TN, while a negative correlation was observed with urease levels. This phylum, characterized by its thick-walled structure, exhibits the ability to generate budding spores as a defense mechanism against detrimental external factors, showcasing a high resistance to stress (Dai et al., 2019). The findings of this study indicate a positive correlation between Proteobacteria and Bacteroidetes with pH and Na, while also revealing a negative correlation with organic matter, which aligns with the results of a previous investigation (Lauber et al., 2009). Bacteroidetes prefer high pH environments for survival, and pH has been considered as a major factor in the formation of bacterial communities (Ganzert et al., 2014). Consequently, the reduction in the abundance of Bacteroidetes can be primarily attributed to the alteration in pH subsequent to the application of compost. This implies that alterations in microbial communities within the soil have the potential to enhance soil characteristics, diminish sodium content in saline-alkali soil, and foster plant growth (Supplementary Figure S1D). The findings of this study further substantiate the hypothesis that the addition of compost to saline soils can enhance soil nutrient levels. The various alterations made to the soil, particularly the changes in pH resulting from compost application, significantly influenced the diversity of the bacterial community in saline soil (Naeem et al., 2018).

Soil enzymes play a crucial role in assessing soil fertility and are actively engaged in the cycling and transformation of soil nutrients, thereby serving as indirect indicators of microbial activity (Jing et al., 2019). The imposition of salinity stress has been found to considerably diminish the activities of soil urease and catalase, while concurrently exerting a detrimental impact on soil protease activity (Azadi and Raiesi, 2021). In the current investigation, the application of compost exhibited a significant enhancement in soil catalase and urease activities, as well as alterations in the abundance of soil bacterial communities when compared to the control group (CK). These findings suggest a substantial increase in soil enzyme activity due to the application of compost (Supplementary Figures S1E,F). This was caused by the interaction between the plant itself and its microorganisms. The augmentation of enzyme activity further substantiates the efficacy of compost product application in ameliorating saline soil conditions (Wang Y. M. et al., 2022). Therefore, cultivating these dominant flora in saline soils and inoculating them into plant roots may improve the microbial diversity in the soil. These microorganisms release some active enzymes, promote the synthesis of organic matter and humus, and release some growth-promoting factors, among other biochemical reactions (Ye et al., 2009), thereby improving the plant growth environment and enhancing saline soil nutrients. The application of this intervention resulted in an improved availability of soil nutrients and enhanced physicochemical properties. Additionally, it led to an increase in both the diversity and activity of soil bacteria, consequently altering the composition of soil bacterial communities. Notably, the presence of Proteobacteria, Firmicutes, Actinobacteriota, and Bacteroidota was found to positively impact the quality of saline-alkali soil.

## Conclusion

This study showed that composting with different proportions of raw materials produced mature end-products and altered the bacterial community succession. This study additionally examined the impacts of incorporating compost as a soil amendment on the fertility and microbial diversity of saline soils. The findings from 16S RNA amplicon sequencing revealed that Proteobacteria, Firmicutes, Actinobacteria, and Chloroflexi were the prevailing bacterial taxa in saline soils. RDA results indicated that pH, TN, organic matter, and soil enzymes were the key parameters affecting the bacterial community. These findings may provide new ideas for compost as a bioamendment for saline soils.

## Data availability statement

The Illumina sequences were deposited in the NCBI Sequence Read Archive (accession numbers SRR24001333–SRR24001347 for the rhizosphere soil bacteria of potted 16S rRNA gene data and SRR24031951– SRR24031993 for the compost rhizosphere soil 16S rRNA gene data).

## Author contributions

DX: Writing – original draft. XY: Methodology, Writing – review & editing. JinC: Methodology, Visualization, Writing – review & editing. XL: Formal analysis, Methodology, Software, Writing – review & editing. JiaC: Conceptualization, Supervision, Writing – review & editing. JL: Conceptualization, Supervision, Writing – review & editing.
