# Supplementary material for: Effects of compost as a soil amendment on bacterial community diversity in saline–alkali soil
Source: Front Microbiol. 2023 Sep 27;14:1253415. doi: 10.3389/fmicb.2023.1253415 (PMC10565496; doi:10.3389/fmicb.2023.1253415)
Supplement: Supplementary file 1 [file Image_1.pdf]

Effects of compost as a soil amendment on bacterial community diversity in saline–alkali soil

Daolong Xu[1], Xiaowen Yu[2], Jin Chen[3], Xiufen Li[4], Jian Chen[1], JiangHua Li[1*]

[1]*Jiangnan Univ, Natl Engn Lab Cereal Fermentat Technol, 1800 Lihu Rd, Wuxi 214122, Jiangsu, Peoples R China.*

[2]*Inner Mongolia Univ, Sch Life Sci, Minist Educ, Key Lab Forage & Endem Crop Biotechnol, Hohhot 010021, Peoples R China.*

[3]*Anhui Agricultural University, College of Life Sciences, 130 Changjiang West Road, Hefei City, China.*

[4]*School of Environment and Civil Engineering, Jiangnan University, 214122 Wuxi, China.*

*Corresponding author Address: Jiangnan Univ, Natl Engn Lab Cereal Fermentat Technol, 1800 Lihu Rd, Wuxi 214122, Jiangsu, Peoples R China.

Tel/Fax: +0510－85329031

E-mail address: lijianghua@jiangnan.edu.cn

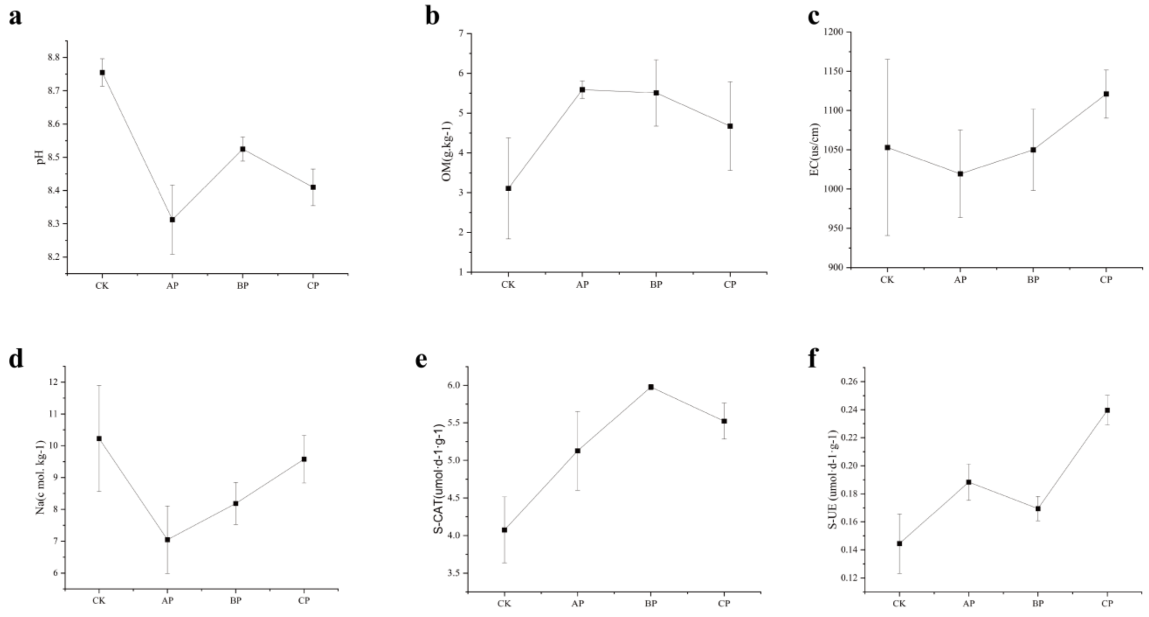

Fig.S1 Effects of adding compost on nutrients in saline-alkali soil. OM: organic matter. EC: electrical-conductivity. Na: Sodium content. MC: moisture content. SCAT: solid catalase. SUE: solid-urease. Different small indicate significant difference between different treatments ($p < 0.05$). The error bars represent the standard error of the mean ($n = 4$).
